# Palivizumab Compliance by Infants in Puerto Rico During the 2009–2010 Respiratory Syncytial Virus Season

**DOI:** 10.1007/s10900-014-9877-z

**Published:** 2014-04-23

**Authors:** Israel Matías, Inés García-García, Lourdes García-Fragoso, Marta Valcárcel

**Affiliations:** Department of Pediatrics, Neonatology Section, UPR School of Medicine, PO BOX 365067, San Juan, PR 00936-5067 USA

**Keywords:** Prophylaxis, Prevention, Bronchiolitis, Viral diseases, Prematurity

## Abstract

Respiratory syncytial virus (RSV) is the leading viral pathogen responsible for bronchiolitis and pneumonia in infants. We assessed palivizumab prophylaxis compliance for infants in Puerto Rico. We retrospectively studied data from 868 infants (409 females, 459 males) during the 2009–2010 RSV season. The infants had a mean gestational age of 33 weeks (range 23–41) and a mean birth weight of 1,767 g (range 509–4,120). Only 74 % of the infants with indications received prophylaxis. The main reasons for noncompliance were non-approval by the medical insurance (53 %), parents’ unavailability (31 %), and infant sickness (11 %). Infants with the government medical insurance were more likely to be denied prophylaxis and to receive fewer doses. Parents need to be educated on the importance of RSV prophylaxis. Physicians should be aware that many infants are not being dosed appropriately and that strategies need to be established to improve compliance.

## Introduction

Respiratory syncytial virus (RSV) is the leading viral pathogen responsible for bronchiolitis and pneumonia in infants during the first year of life in the United States. The risk of serious RSV illness is highest among those with prematurity, bronchopulmonary dysplasia (BPD), chronic lung disease (CLD), congenital heart disease (CHD), congenital abnormalities of the airway or neuromuscular disease, and certain immunodeficiencies. Eighty to ninety percent of infant hospitalizations are RSV-related and the majority of these occur in children younger than 6 months of age [1].

The prevention of this infection is available with intramuscular humanized monoclonal antibody (palivizumab). Palivizumab has been found to be safe in doses of 15 mg/kg administered by intramuscular injection every 28–30 days during the RSV season. In 1998, the results of a multicenter, multinational, phase III trial (IMpact-RSV) to evaluate the safety and effectiveness of monthly administration of palivizumab as prophylaxis for serious RSV illness in high-risk infants was published. Palivizumab reduced the incidence of hospitalization due to RSV compared to placebo by 55 % [2, 3]. Children randomized to palivizumab had fewer days of RSV hospitalization and fewer days of supplemental oxygen therapy. The authors reported that intramuscular injections were well tolerated and there were no toxicities associated [3].

In Puerto Rico, RSV infections are seen throughout the year with a peak season starting in July and ending in March. In 2009, the American Academy of Pediatrics (AAP) published new guidelines for RSV prophylaxis recommending fewer doses for premature infants born at 32–35 weeks of gestation in all geographical areas. Recommendations for initiation and termination of prophylaxis were modified to reflect current descriptions from the Centers for Disease Control and Prevention (CDC) of RSV seasonality in different geographic locations within the United States. Regardless of the month in which the first dose is administered, the recommendation for a maximum number of 5 doses for all geographic locations is emphasized for infants with hemodynamically significant CHD, CLD, or birth before 32 weeks’ 0 days’ gestation. A maximum number of three doses were recommended for infants with a gestational age of 32 weeks 0 days to 34 weeks 6 days without hemodynamically significant CHD or CLD who qualify for prophylaxis [4]. Due to the year round prevalence of RSV in Puerto Rico infants in the island were eligible to receive up to nine doses of palivizumab during the RSV season. After the new AAP statement was released, the Puerto Rico Health Department made the resolution to follow their recommendations, and decreased the number of doses given.

We studied the data of all the infants who received RSV prophylaxis during the 2009–2010 RSV season in Puerto Rico, distributed by one specific specialty pharmacy with the objectives of assessing if infants at risk of RSV infection were receiving palivizumab as recommended by the 2009 AAP guidelines and evaluating the reasons for noncompliance.

## Materials and Methods

We retrospectively analyzed deidentified data from a cohort of patients receiving palivizumab. The data was collected from the Special Care Pharmacy and Compounding Services database for patients who were eligible to receive RSV prophylaxis during the 2009–2010 RSV season in Puerto Rico (from July 2009 to March 2010). Special Care Pharmacy was the main distributor of palivizumab in Puerto Rico providing services to approximately 90 % of the infants referred for RSV prophylaxis. They collected data prospectively during the season which was made available to us without identifiers. Data included demographics, eligibility for palivizumab, medical insurance approval, number of doses received and interval between doses. Subjects from all over the island and from every medical insurance, private and government were included.

The statistical analysis of collected data was performed by using frequency, means, median, and ranges. Differences among groups were evaluated using Chi square and *t* test. A *p* value <0.05 were considered statistically significant. The study was approved by the University of Puerto Rico Medical Sciences Campus Institutional Review Board.

## Results

**Fig. 1:**
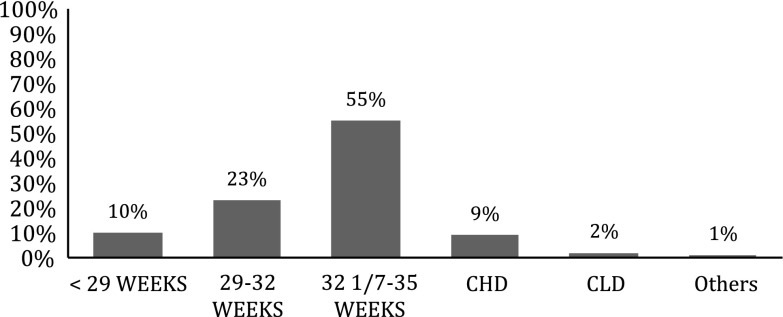
Distribution of risk groups eligible to receive palivizumab in the studied population (N = 868)

The data of 868 infants (47 % females, 53 % males) who qualified to receive RSV prophylaxis during the 2009–2010 RSV season were evaluated. The mean gestational age of the infants was 33 weeks (range 23–41), and the mean birth weight was 1,767 g (range 509–4,120). Figure 1 shows the distribution of subjects by risk group who were eligible to receive palivizumab prophylaxis. Most of them (55 %) were premature infants born at 32 1/7–35 weeks of gestation followed by those born at 29–32 weeks of gestation. Infants were equally distributed with regard to government (public) medical insurance (50 %) and private insurances (50 %).

Seventy-four percent of the infants (n = 640) referred to the specialty pharmacy received at least one dose of palivizumab prophylaxis in the studied season. The median number of doses administered was 2 (range 1–9), and the median interval between the doses was 32 days (range 23–123). The total number of palivizumab doses received by all infants was 1,163 and 69 % of the doses (n = 806) were administered in intervals not >35 days. Six percent of the doses (n = 68) were administered in intervals of <28 days.

Of those patients that qualified to receive a maximum of five doses, infants born at <32 weeks of gestation and those with CHD received a median of three doses. Infants with CLD received a median of 4 doses. Of those patients that qualified to receive a maximum of three doses, infants born at 32 1/7–35 weeks of gestation received a median of two doses. The difference in maximum doses between these groups was significant (*p* < 0.01). The maximum number of five doses was received by 27 % of infants born at <32 weeks of gestation, 31 % of infants with CHD, and 47 % of those with CLD. The maximum number of three doses was received by 36 % of infants born at 32 1/7–35 weeks of gestation. Only 16 % of the patients received their first dose before discharge from the hospital. The reasons for noncompliance with the recommended regimen of RSV prophylaxis were non-approval by the medical insurance (53 %) followed by unavailability of the parents (31 %), sick infant (11 %), not being able to afford co-pay (3 %), lack of transportation (1 %), and no interest in the prophylaxis (1 %).

**Fig. 2:**
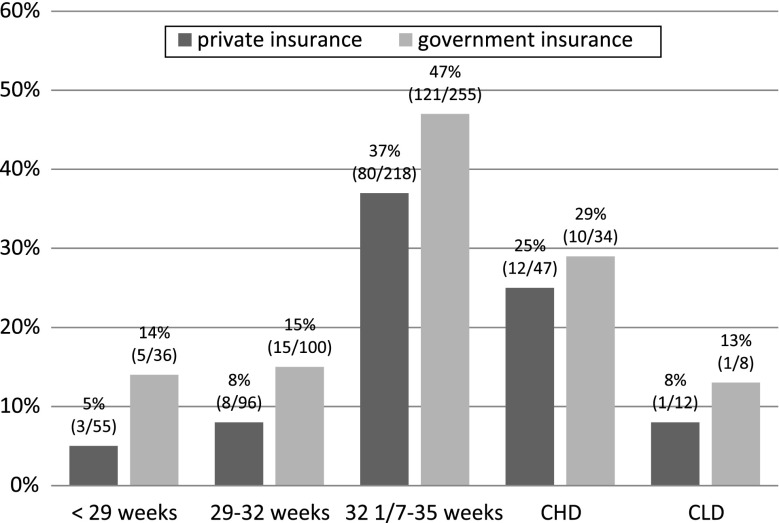
Palivizumab coverage denial by eligible risk group and medical insurance

After the implementation of the 2009 AAP guidelines, infants born at 32 1/7–35 weeks of gestation were more likely to be denied prophylaxis (*p* < 0.01). RSV prophylaxis was not approved by medical insurance in 9 % of the infants born at <29 weeks of gestation, 12 % of the infants 29–32 weeks of gestation, 42 % of the infants 32 1/7–35 weeks of gestation, 28 % of the infants with CHD, and 10 % of the infants with CLD. Figure 2 shows medical insurance coverage denials by risk group and medical insurance. Infants with government medical insurance were more likely to be denied prophylaxis (36 % denials vs. 24 % denials, *p* < 0.01) and to receive fewer doses (two doses vs. three doses, *p* < 0.01).

## Discussion

Palivizumab prophylaxis has been shown to reduce the incidence of RSV hospitalization in at-risk infants [3, 5], but effective prophylaxis requires full compliance with the monthly dosing schedule [6]. Noncompliance is common and may be the most controllable barrier to pharmacologic prevention of RSV infection and its potentially severe sequelae in high-risk infants [7]. Studies analyzing compliance rates of palivizumab, defined in various ways across the studies, have shown rates as low as 25 % to as high as 100 % [8–10]. The compliance rates of patients in clinical studies demonstrating reduced hospitalization rates in high risk infants involved in palivizumab licensure were 92 and 93 % [3, 11]. Thus, compliance in routine practice was more variable. In our study 74 % of the infants received at least one dose of palivizumab, and <50 % received the recommended maximum number of doses, much less than in studies that demonstrated efficacy of palivizumab.

Berger and colleagues assessed palivizumab use in 10,390 infants based on dispensing records for a pharmacy benefits management company that provided follow-up telephone contact to ensure the prescribed dose was administered. A total of 9,675 (93 %) of 10,390 infants were found to be compliant, defined as having no more than 35 days between shipment of doses [12].

RSV hospitalization rates were 1.4 % in the compliant group versus 3.1 % in the noncompliant group (OR 2.2, 95 % CI 1.4–3.5, *p* < 0.001) [12]. Frogel and coworkers also examined compliance and RSV hospitalization rates, suggesting that improved compliance was associated with a reduced risk of RSV hospitalization, consistent with the clinical efficacy of palivizumab [13]. These studies show that despite the progress that has been made in reducing RSV hospitalization rates, infants whose parents are noncompliant with palivizumab continue to have higher hospitalization rates [12]. Our study shows high rates of noncompliance. However, due to the nature of the study design no clinical outcome data of the patients were available and noncompliance cannot be correlated with worse outcomes.

A systematic review of compliance with palivizumab administration showed that the most common barriers that influence or predict noncompliance with the recommended regimen of RSV prophylaxis were parental smoking, Medicaid enrollment, lower parental expectations for the benefits of RSV prophylaxis, lack of transportation, and language difficulties [14]. Our study showed similar results with respect to medical insurance where infants with government medical insurance were more likely to be denied prophylaxis and to receive fewer doses. This finding needs attention since palivizumab, when dosed consistent with Food and Drug Administration (FDA) approved labeling has been shown to be cost-effective among infants enrolled in Medicaid [15].

The support services provided by specialty pharmacies are now widely used to streamline the drug distribution, delivery, and management process in ways that engage patients in their care [16]. In a recently published article the patients who were eligible to receive palivizumab prophylaxis had a 18 % higher likelihood of receiving the 2009 AAP recommended doses when they were administered through a specialty pharmacy rather than a traditional pharmacy. The analysis found that 83 % of infants who obtained their palivizumab from a specialty pharmacy received the recommended doses, compared to 66 % who received their medication from a non-specialty pharmacy, thus emphasizing how specialty pharmacies can improve patient compliance and treatment course completion [17]. In Puerto Rico, specialty pharmacies have distributed, managed insurance approvals, and delivered palivizumab since RSV prophylaxis started in 1999, but compliance remains poor. The reasons for this are unclear and should be investigated.

A procedure involving extensive counseling of parents, reminder telephone calls on the day prior to the appointment, calendars with reminder stickers, and tracking charts in the nursing medication rooms was used to improve compliance in a hospital-based clinic. Results showed that an increased percentage of infants (71 %) received the appropriate number of injections after implementation of the new interventions compared with 25 % before the new interventions [8]. Some authors have reported that a home care strategy for administration of palivizumab was associated with better compliance with therapy by offering consistent delivery of medication and ongoing parent/caregiver education. Based on these studies, a home-based program to administer RSV immunoprophylaxis was proposed as a key component in ensuring compliance during the RSV season. Most barriers that affect compliance could be overcome, providing greater opportunity to educate parents or caregivers on the risks of severe RSV disease and the benefits of prophylaxis. A home-based delivery system might offer some additional benefit of decreasing exposure of the infant to pathogens, including RSV, in the clinic or office setting [14]. A strategy like this one may help Puerto Rican infants improve their compliance by eliminating the intermediary between the specialty pharmacy and the patient. At present, the specialty pharmacies deliver prophylaxis to the pediatricians’ office and they administer it. With this approach doses may be missed or administered at longer intervals as they depend on the parents taking the child to the office for administration. The development of a reminder system may also help improve compliance.

It is critically important that parents of children at high risk for severe RSV disease be empowered with clear information about the seriousness of RSV disease and the benefits of palivizumab prophylaxis to enable them to make informed choices [6]. Physicians and other health professionals are primary sources of information for patients or their caregivers, and play a key role in guiding families in their choices about RSV prophylaxis and may play an important role in improving compliance [6, 18].

## Conclusions

Our study emphasizes the need to educate parents on the serious consequences of RSV infection in high-risk patients and the importance of administration of prophylaxis in the recommended interval during the RSV season to prevent RSV hospitalizations and their complications. Physicians should be aware that many infants are not being dosed appropriately and that strategies need to be established in order to improve compliance. These strategies should include working with payors to ensure that all infants who qualify for prophylaxis are given approval for its use.
